# Supplementary material for: Repetitive Transcranial Magnetic Stimulation (rTMS) of Dorsolateral Prefrontal Cortex May Influence Semantic Fluency and Functional Connectivity in Fronto-Parietal Network in Mild Cognitive Impairment (MCI)
Source: Biomedicines. 2022 Apr 25;10(5):994. doi: 10.3390/biomedicines10050994 (PMC9138229; doi:10.3390/biomedicines10050994)
Supplement: Supplementary file 1 [file biomedicines-10-00994-s001.zip › biomedicines-1673799-supplementary.pdf]

Supplementary Table S1. Between-group comparison on comprehensive cognitive and behavioral measures at pre-treatment evaluation via Kruskal-Wallis test (*H*) and Chi-Square test ($\chi2$); post-hoc analyses were executed by Mann-Whitney test.

Variable	MCI-TMS (*n*= 11)	MCI-C (*n*= 16)	HC (*n*=13)	H / χ^2	*p*-value	Adj-*p*	Benjamini-Hochberg adjusted post-hoc analyses		
							MCI-TMS vs MCI-C	MCI-TMS vs HC	MCI-C vs HC
Cognitive assessment:<br>*RBANS subtests:*									
List Learning	21.00<br>(16.00, 24.00)	17.50<br>(15.25, 21.50)	26.00<br>(23.00, 28.50)	15.01	0.001	**0.001**	0.250	**0.010**	**<0.001**
Story Memory-IR	15.00<br>(14.00, 17.00)	13.00<br>(9.25, 14.50)	20.00<br>(18.00, 22.50)	20.61	<0.001	**<0.001**	0.050	**<0.001**	**<0.001**
Figure Copy	15.00<br>(12.00, 17.00)	12.00<br>(10.25, 13.75)	15.00<br>(12.50, 16.00)	6.48	0.039	0.053	-	-	-
Line Orientation	14.00<br>(11.00, 17.00)	14.00<br>(11.25, 16.50)	17.00<br>(14.50, 18.50)	4.13	0.126	0.135	-	-	-
Picture Naming	10.00<br>(10.00, 10.00)	9.00<br>(9.00, 10.00)	10.00<br>(10.00, 10.00)	12.03	0.002	0.135	-	-	-
Semantic Fluency	13.00<br>(10.00, 15.00)	15.00<br>(13.25, 17.00)	19.00<br>(16.50, 21.00)	16.96	<0.001	**<0.001**	**0.044**	**<0.001**	**0.001**
Digit Span	6.00<br>(6.00, 8.00)	8.00<br>(6.00, 9.75)	9.00<br>(8.00, 11.50)	6.70	0.035	0.052	-	**-**	-
Coding	25.00<br>(16.00, 33.00)	20.00<br>(14.75, 29.75)	39.00<br>(30.50, 45.50)	13.00	0.001	**0.003**	0.543	**0.010**	**<0.001**
List Recall	1.00<br>(0.00, 3.00)	0.50<br>(0.00, 1.00)	4.00<br>(2.50, 6.50)	12.76	0.002	**0.003**	0.511	**0.032**	**<0.001**
List Recognition	13.00<br>(13.00, 18.00)	16.00<br>(15.00, 16.75)	18.00<br>(17.50, 20.00)	17.07	<0.001	**<0.001**	0.250	**0.002**	**<0.001**
Story Recall-DR	1.00<br>(0.00, 7.00)	5.00<br>(3.25, 7.00)	10.00<br>(9.00, 11.50)	22.69	<0.001	**<0.001**	0.317	**<0.001**	**<0.001**
Figure Recall	3.00<br>(0.00, 7.00)	3.50<br>(2.00, 5.00)	10.00<br>(7.50, 11.50)	15.75	<0.001	**0.001**	0.511	**0.002**	**<0.001**

To be continued

Supplementary Table S1. Continued.

Variable	MCI-TMS (*n*= 11)	MCI (*n*= 16)	HC (*n*=13)	H / χ^2	*p*-value	Adj-*p*	Benjamini-Hochberg adjusted post-hoc analyses		
							MCI-TMS vs MCI	MCI-TMS vs HC	MCI vs HC
Behavioural measures:									
Beck Depression Inventory-II	15.00 (3.00, 21.00)	12.00 (6.50, 16.50)	6.00 (2.00, 9.50)	5.14	0.077	0.095	-	-	-
Beck Anxiety Inventory	3.00 (2.00, 10.00)	6.50 (2.75, 13.75)	6.00 (2.50, 6.50)	0.78	0.675	0.675	-	-	-
Apathy Evaluation Scale	38.00 (33.00, 42.00)	35.50 (28.50, 40.00)	31.00 (24.50, 32.50)	8.61	0.013	**0.022**	0.394	**0.009**	0.060

Note. MCI-TMS, patient with Mild Cognitive Impairment and underwent to TMS; MCI-C, patients with Mild Cognitive Impairment and not underwent to TMS; HC, healthy controls; Adj-*p* represents *p*-value corrected for multiple comparisons using Benjamini–Hochberg procedure; statistically significant differences are shown in **bold**.

Supplementary Table S2. Between-group comparison on comprehensive cognitive and behavioral measures at post-treatment evaluation (using pre-treatment scores as covariates) via Quade's rank analysis.

Variable	MCI-TMS (*n*= 11)	MCI (*n*= 12)	HC (*n*=12)	Quade's test	*p*-value	Adj-*p*	Benjamini-Hochberg adjusted post-hoc analyses		
							MCI-TMS vs MCI	MCI-TMS vs HC	MCI vs HC
Cognitive assessment:									
RBANS subtests:									
List Learning	22.00 (19.00, 28.00)	20.00 (17.25, 22.25)	25.00 (22.00, 28.50)	0.06	0.935	0.957	-	-	-
Story Memory-IR	14.00 (9.00, 17.00)	11.00 (9.25, 14.50)	17.00 (15.00, 19.50)	0.20	0.814	0.957	-	-	-
Figure Copy	14.00 (10.00, 16.00)	11.00 (10.00, 12.00)	14.00 (13.00, 16.00)	1.60	0.214	0.459	-	-	-
Line Orientation	15.00 (14.00, 16.00)	12.00 (10.00, 14.00)	18.00 (14.50, 20.00)	7.27	0.002	**0.031**	**0.014**	0.521	**0.002**
Picture Naming	10.00 (9.00, 10.00)	9.00 (9.00, 10.00)	10.00 (10.00, 10.00)	3.87	0.030	0.148	-	-	-
Semantic Fluency	10.00 (6.00, 13.00)	8.00 (6.25, 8.75)	13.00 (10.50, 15.50)	6.35	0.004	**0.031**	**0.026**	0.487	**0.005**
Digit Span	8.00 (6.00, 10.00)	8.00 (6.00, 9.75)	9.00 (7.50, 11.50)	0.22	0.800	0.957	-	-	-
Coding	28.00 (16.00, 35.00)	27.00 (16.00, 31.00)	42.00 (31.00, 47.50)	0.30	0.737	0.957	-	-	-
List Recall	0.00 (0.00, 3.00)	2.00 (0.00, 3.00)	5.00 (2.00, 5.00)	1.63	0.209	0.459	-	-	-
List Recognition	16.00 (13.00, 17.00)	16.00 (14.25, 17.75)	18.00 (16.00, 19.00)	0.11	0.890	0.957	-	-	-
Story Recall-DR	3.00 (0.00, 7.00)	5.00 (5.00, 6.00)	8.00 (7.50, 10.50)	1.23	0.302	0.567	-	-	-
Figure Recall	1.00 (0.00, 10.00)	6.00 (4.00, 9.00)	9.00 (6.50, 12.00)	2.69	0.081	0.243	-	-	-

To be continued

Supplementary Table S2. Continued.

Variable	MCI-TMS (*n*= 11)	MCI (*n*= 12)	HC (*n*=12)	Quade's test	*p*-value	Adj-*p*	Benjamini-Hochberg adjusted post-hoc analyses		
							MCI-TMS vs MCI	MCI-TMS vs HC	MCI vs HC
Behavioural measures:									
Beck Depression Inventory-II	10.00 (4.00, 15.00)	9.00 (8.00, 16.25)	4.50 (3.00, 9.25)	0.40	0.669	0.957	-	-	-
Beck Anxiety Inventory	3.00 (2.00, 25.00)	3.50 (3.00, 12.75)	3.50 (2.00, 9.25)	0.04	0.957	0.957	-	-	-
Apathy Evaluation Scale	37.00 (35.00, 39.00)	35.00 (29.50, 39.50)	28.00 (24.00, 31.50)	3.09	0.057	0.213	-	-	-

Note. MCI-TMS, patient with Mild Cognitive Impairment and underwent to TMS; MCI, patients with Mild Cognitive Impairment and not underwent to TMS; HC, healthy controls; Adj-*p* represents *p*-value corrected for multiple comparisons using Benjamini–Hochberg procedure; statistically significant differences are shown in **bold**.
